# Brain medical image diagnosis based on corners with importance-values

**DOI:** 10.1186/s12859-017-1903-6

**Published:** 2017-11-21

**Authors:** Linlin Gao, Haiwei Pan, Qing Li, Xiaoqin Xie, Zhiqiang Zhang, Jinming Han, Xiao Zhai, the Alzheimer’s Disease Neuroimaging Initiative

**Affiliations:** 1 Research center for intelligent information processing, College of Computer Science and Technology, Harbin Engineering University, Harbin, 150001 China; 2 Department of Computer Science, City University of Hong Kong, Kowloon, Hong Kong; 3 Department of Neurology and Neuroscience Center, the First Hospital of Jilin University, Changchun, 130021 China

**Keywords:** Brain medical image diagnosis, Corner detection, Multilayer texture images, Corner matching, Bipartite graph, Classification

## Abstract

**Background:**

Brain disorders are one of the top causes of human death. Generally, neurologists analyze brain medical images for diagnosis. In the image analysis field, corners are one of the most important features, which makes corner detection and matching studies essential. However, existing corner detection studies do not consider the domain information of brain. This leads to many useless corners and the loss of significant information. Regarding corner matching, the uncertainty and structure of brain are not employed in existing methods. Moreover, most corner matching studies are used for 3D image registration. They are inapplicable for 2D brain image diagnosis because of the different mechanisms. To address these problems, we propose a novel corner-based brain medical image classification method. Specifically, we automatically extract multilayer texture images (MTIs) which embody diagnostic information from neurologists. Moreover, we present a corner matching method utilizing the uncertainty and structure of brain medical images and a bipartite graph model. Finally, we propose a similarity calculation method for diagnosis.

**Results:**

Brain CT and MRI image sets are utilized to evaluate the proposed method. First, classifiers are trained in N-fold cross-validation analysis to produce the best *θ* and *K*. Then independent brain image sets are tested to evaluate the classifiers. Moreover, the classifiers are also compared with advanced brain image classification studies. For the brain CT image set, the proposed classifier outperforms the comparison methods by at least 8% on accuracy and 2.4% on F1-score. Regarding the brain MRI image set, the proposed classifier is superior to the comparison methods by more than 7.3% on accuracy and 4.9% on F1-score. Results also demonstrate that the proposed method is robust to different intensity ranges of brain medical image.

**Conclusions:**

In this study, we develop a robust corner-based brain medical image classifier. Specifically, we propose a corner detection method utilizing the diagnostic information from neurologists and a corner matching method based on the uncertainty and structure of brain medical images. Additionally, we present a similarity calculation method for brain image classification. Experimental results on two brain image sets show the proposed corner-based brain medical image classifier outperforms the state-of-the-art studies.

## Background

Image-based brain disorder diagnosis has attracted increasing interest in computer-assisted interventions [1]. This study has been handled efficiently with the application of machine learning methods [2]. Among numerous machine learning methods, deep learning has been showing the state-of-the-art performance in the recent years. It has been applied in many fields of computer vision, natural language process, and medical image analysis. However, deep learning is still limited because of its vast number of network parameters that must be learned from a large amount of data. It is noteworthy that data sets collected in brain imaging studies are commonly very small. Thus deep learning is still a challenging method in brain image analysis. In the prevalent framework of machine learning for brain imaging data diagnosis, feature extraction is an essential step [1]. Rong et al. have proposed a symmetry-based brain CT image classification method consisting of three stages [3]. First, the weak symmetry of the histograms of two hemispheres is used to classify brain CT images into normal or abnormal categories. Second, the strong symmetry of the textures between two hemispheres in abnormal images are extracted to locate lesions. At last, the abnormal images are classified into benign or malignant ones based on the features of lesions. A few caveats exist in this method. For instance, for abnormal images with similar lesions in both hemispheres or with lesions in the middle of brain, the weak or strong symmetry features are insufficient to distinguish normal and abnormal images. Ding et al. have proposed a joint feature selection method from voxel-based morphometry (VBM) and texture analysis to distinguish Alzheimer’s disease (AD) from the normal controls [4]. However, this method is not stable for brain disease diagnosis using brain medical images with different intensity ranges.

Corners are the small points of interest with variation in two dimensions [5]. They are essential features of images and play a critical role in grasping objects. Tremendous progress has been made over corner research [5–8], but many fundamental problems are still open, such as corner detection, matching, and corner-based image classification.

As a fundamental and important step in many vision tasks such as image matching, recognition and tracking [5, 6, 9], corner detection has been well studied for many years. Existing methods are divided into three main categories: gray intensity-based, contour-based, and model-based methods [10]. One of the most classical gray intensity-based methods is the Harris method [11]. It is broadly applied in many cases due to its advantages of rotation, translation, and illumination invariance [10]. Nevertheless, Harris is prone to produce numerous useless corners when used over a whole grayscale image. In the field of medical image analysis, doctors generally pay considerable attention to the morphology of ROIs (Regions of Interest). Thus, corners detected from those uninteresting regions are prone to be useless. Moreover, Harris only stores the coordinates of corners, leading to the loss of important medical domain knowledge. Concretely, these corners do not preserve the uncertainty and structure of pixels in brain medical images [12]. To address the above inadequacy, contour-based corner detection methods [13–15] might be considered. This type of methods first extracts curves of images using edge detectors, and then searches for the curvature maxima along those curves as corners. Though these methods detect corners on image contours to reduce useless corners, the extracted contours are regarded equally. This does not integrate doctors’ diagnostic information. Thus, the detected corners lose the essential domain knowledge. In terms of the model-based methods, existing methods are required to fit images into a predefined model. These methods are often limited to specific types of points [16]. Moreover, these methods are time-consuming and detected corners do not have the significant diagnostic information either.

Corner matching is to realize the correspondences of two corner sets by estimating the transformation from a corner set to the other [17]. Existing corner matching studies mainly fall into two categories: rigid and non-rigid. Rigid methods are mainly developed based on the classical iterative closest point (ICP) [18], which uses affine transformations to find the closest corresponding points between two surfaces. Nonetheless, methods based on the ICP do not fully capture the anatomical variability, especially comparing shape with significant differences [19]. Thus, rigid methods are less applicable in real-world problems [20]. Non-rigid corner matching methods generally produce an initial matched corner pair sequence first. Then these methods evaluate the transformation between initial matched corner pair sequences to generate a final matched corner pair sequence. Yi et al. [17] have used *l*_1_-norm to formalize the transformation between initial matched corner pair sequences. However, they have not argued the generation of initial matched corner pair sequences. Thus, the uncertainty of corners is not taken into account in the corner matching process. Myronen and Song [20] have proposed a probabilistic method to realize the alignment between corner sets. They regard corners as a group and move a group of corners coherently to keep the topological structure among corners. Since the deformations of human brains usually happen in local regions, coherent movement causes improper matching among corners from other regions. This would lead to the failure of the generation of the best correspondence. Thus, this method is inappropriate for corner matching in brain medical images. Moreover, the above corner matching methods are used for 3D image registration. They are to match corners of the images from the same object or person, unlike the corner matching of the brain medical images from different patients.

To address these problems, an automatic multilayer texture image (MTI) extraction method is first proposed for corner detection. The method integrates the diagnostic knowledge from neurologists and assigns each corner an importance-value representing the uncertainty of a pixel. Second, a corner matching method is presented combining the uncertainty and structure of brain medical images. It generates an initial matched corner pair sequence based on the definition of matched corner pairs and utilizes a bipartite graph model to reduce the redundancy of the initial matched corner pair sequence. At last, a similarity function is proposed based on the matched and unmatched corner pairs to make diagnoses for brain medical images. Experimental results on brain CT and MRI image sets show that the proposed corner-based brain medical image diagnosis method outperforms three state-of-the-art methods on accuracy and F1-score. Additionally, it is more robust to different intensity ranges of brain medical image.

## Methods

### Workflow

**Fig. 1:**
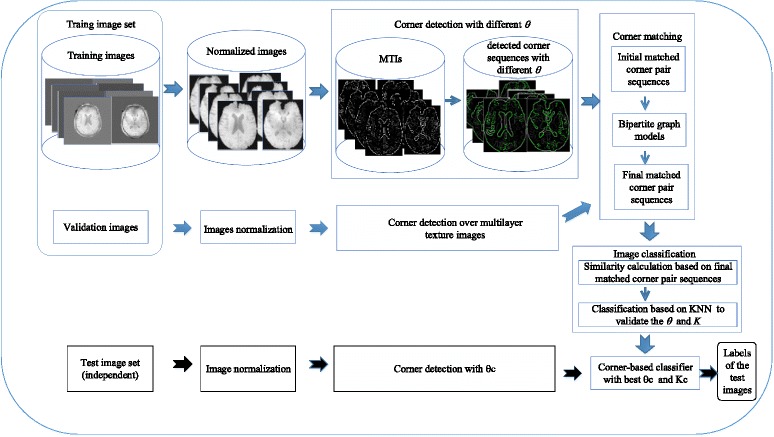
Workflow of the analysis. The sequence with the blue arrows depicts the training of the classifier with the corner response threshold *θ* in corner detection and with the *K* in the *K*-nearest neighbor model. The other sequence with the black arrows aims to test the trained classifier.

The workflow is schematically illustrated in Fig. 1. It comprises two parts. The first part is to train a classifier with blue arrows as guidance. A set of labeled brain medical images are divided into training and validation images to train the classifier. First, each image is normalized into uniform size. Second, each normalized image generates a MTI and corners with importance-values are detected on the MTI under a specific *θ*. Third, each group of corner sequences, one from the validation images and the other from the training images, is matched to generate an initial matched corner pair sequence. The initial matched corner pair sequence is transformed to a bipartite graph to yield the final matched corner pair sequence. After that, the similarity between validation and training images is determined based on their final matched corner sequence. The labels of validation images are predicted based on the KNN model to validate the values of *θ* and *K*. Finally, the best θc of *θ* and Kc of *K* are utilized to construct the classifier. From the above steps, a corner-based classifier with θc and Kc is generated. In the second part (with black arrows as guidance), given a test image set, after preprocessing, corners are detected and matched with the corners of the training images. Then, the labels of testing images can be predicted based on the trained classifier. Table 1 displays the frequently used symbols.

**Table 1:** Frequently used symbols

Symbols	Meaning
*I*	An original grayscale image
GI	Normalized grayscale image
*Row*	The number of the rows in GI
*Column*	The number of the columns in GI
MTI	Multilayer texture image
*θ*	A corner response threshold
*c*	A corner
(*m,n*)	A corner that is located in the *m*th row and *n*th column
*C*	A corner sequence
*Mov*(*m*, *n*)	Mobility of the corner (*m, n*)
[*ci*, *cj*]	A matched corner pair
*CI*	An initial matched corner pair sequence
*CM*	The final matched corner pair sequence
*K*	The *K* of the KNN model

### Brain medical image sets

**Fig. 2:**
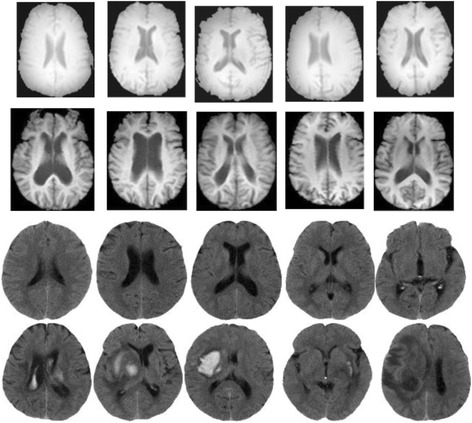
Examples of “Normal” and “Abnormal” images. Brain MRI images in the first row belong to “Normal” category and that in the second row are “Abnormal” ones. Brain CT images in the third row are “Normal” and that in the fourth row are “Abnormal”

Two brain medical image sets are used in this study. The first one consists of 500 brain CT images and is denoted as Dct (Images are detailed in Additional file 1). It is made up of 3 types of images: 380 normal, 92 cerebral infarction (CI), and 28 cerebral hemorrhage (CH) images. These images are divided into two categories: “Normal” and “Abnormal”. The 380 normal images belong to the “Normal” category and the others comprise the “Abnormal” one. Each image in Dct has 256 grayscale values and is labeled by specialists from a neurological department. The second image set is made of 850 brain MRI images and is denoted as the Dmri. These images are downloaded from ADNI (http://adni.loni.usc.edu/). The ADNI was launched in 2003 as a public-private partnership, led by Principal Investigator Michael W. Weiner, MD. The primary goal of ADNI has been to test whether serial MRI, positron emission tomography (PET), other biological markers, and clinical and neuropsychological assessment can be combined to measure the progression of mild cognitive impairment (MCI) and early Alzheimer’s disease (AD). For up-to-date information, see www.adni-info.org. The downloaded Dmri contains two categories of MRI images: 430 normal and 420 AD images. Each image in Dmri has 65536 grayscale values. Additionally, all of the images in Dct and Dmri contain the ventricle portion. Figure 2 shows some “Normal” and “Abnormal” image examples.

### Brain medical image normalization

Original brain medical images are usually produced in different sizes and angles and also display some useless information. These original brain images consist of three portions: background, skull and the intracranial portion. Background is useless for doctors’ diagnosis and always contains noise. Moreover, when doctors make diagnoses, they usually focus on the intracranial portion. Thus, these original images need to be preprocessed as follows: extracting the intracranial portion using the Canny operator [21] because of the clear disparity of the grayscale values between the skull and the intracranial portion, rotating the intracranial portion into the vertical direction using the method in [22], cropping the image based on the vertical external matrix of its intracranial portion and unifying image size into *Row* ×*Column*, where *Row*/*Column* is the mean of all the row/column numbers of the brain medical image set. Counting the image size of the two brain medical image sets seperately, we normalize all brain CT images into 285×260 and all the brain MRI images into 175×141.

**Fig. 3.**
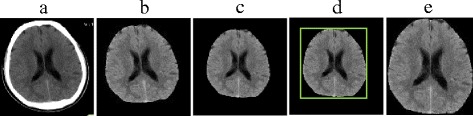
Example of normalizing a brain CT image **a** Original image *I*. **b** Image with the extracted intracranial portion. **c** Rotated image. **d** Image with its vertical external matrix. **e** Normalized grayscale image *GI*.

Figure 3 is an example of normalizing a brain CT image. Figure 3a is an original brain CT image with background and skull. Its intracranial portion is extracted as shown in Fig. 3b. Then its angle is rectified into roughly vertical direction shown in Fig. 3c. After that, the rectified image is cropped and unified into *Row*×*Column* size. The final normalized grayscale image GI is displayed in Fig. 3e, where GI(*m*, *n*) is the grayscale value of the pixel located in (*m*, *n*).

### Corner detection based on multilayer texture images

In the diagnostic process of brain medical images, doctors generally pay more attention to the morphology of the hypodense (dark) and hyperdense (bright) regions than that of other regions [23]. That means different regions in brain medical image embody diverse diagnostic information. Thus, corners detected from these more hypodense and hyperdense regions are more essential than those detected from other regions. Under this guidance, a multilayer texture image (MTI) is first proposed to distinguish the significance of regions in brain medical images. Then, corners are detected on the MTI and are assigned different importance-values to show their significance. Thus, corners detected from MTIs employ certain diagnostic information.

Given a *Row*×*Column* GI, its MTI is comprised of multiple layers of textures. Each layer of textures have the same pixel value. The pixel values denote the significance of pixels and the pixel value and the significance of pixels have a direct correlation. The MTI extraction method is similar to the Canny method [20]. It contains smoothing an image, discovering its intensity gradient, determining the hysteresis double thresholds and generating edges based on the pair of double thresholds. However, the Canny method can only generate a binary texture image by using one pair of double thresholds, and the binary texture image cannot describe the significance of different regions. To handle this, we propose an automatic MTI generation method by using multiple pairs of double thresholds based on the intensity gradient of a GI.

The morphology of the hypodense and hyperdense regions is reflected by the pixels with larger variations in a GI, *i.e*., the pixels with larger intensity gradient values. Moreover, the clearness degree of the morphology of a region has a positive relation to the intensity gradient values of pixels. Thus, the intensity gradient values of a GI are utilized to generate the MTI of the GI. First, pixels of the GI are ordered in descending way based on the intensity gradient values of the GI. Then, the number of these ordered pixels is accumulated to generate a sequence of pairs of double thresholds. Specifically, the histogram *H* of the intensity gradient values of a GI is calculated with *Bn* bins, where *H*(*j*) is the number of pixels in the *j*th bin. If

1 $$ \underset{s^i\in \left\{1,\kern0.5em 2,\kern0.5em \dots, Bn\right\}}{\arg \kern0.2em \min }{\sum}_{j=1}^{s^i}H(j)\ge {R}^i\times Row\times Column, $$

then

2 $$ {highTh}^i={s}^i/ Bn,\kern0.75em {lowTh}^i= rTh\times {highTh}^i. $$

[*highTh* ^*i*^, *lowTh* ^*i*^] is the *i*th pair of double thresholds and is used to extract textures in the *i*th layer.

In Eqs. 1 and 2, *s* ^*i*^ represents the index value of *H*(*j*) and its maximum value is *Bn*. The histogram value of each bin is the number of pixels with several continuous intensity gradient values. Provided the maximum intensity gradient value of an image is *M*, each bin counts the number of pixels with *M*/*Bn* continuous intensity gradient values. *R* ^*i*^ is a decimal iterating from *R* ^*1*^ to 0 with 0.1 drop. It controls the ratio of pixel number whose index gradient values are ordered in descending way. For each *R* ^*i*^, Eq. 1 returns a *s* ^*i*^ value. This indicates that the corresponding pixels in the first *s* ^*i*^ bins of *H*(*j*) have clearer edges than that in the rear bins. Thus, *s* ^*i*^ dividing the maximum bin number *Bn* is used to generate the thresholds, as shown in Eq. 2.

In our study, the *Bn* and *rTh* are set to 64 and 0.3, respectively. Generally, the morphology of the ventricles is the clearest part in a brain medical image. Based on experiments, *R* ^*1*^ is set to 0.8 to produce the first pair of double thresholds [*highTh* ^1^, *lowTh* ^1^], which is used to extract the first layer of textures containing the edges of ventricles. Then *R* ^*i*^ is decreased by 0.1 each time until 0 to produce the remaining pairs of double thresholds. Next these pairs of double thresholds are used to extract the remaining (*N*-1) layers of textures.

It is notable that the pairs of double thresholds are generated in a descending order, and the preceding thresholds are used to extract the textures of clearer regions. Based on this, a MTI is created by setting *MTI*(*m*, *n*)=1/*i* when textures in the *i*th layer pass the pixel (*m*, *n*) yet textures in the preceding (*i*-1) layers do not pass (*m*, *n*). When no texture passes a pixel, that means the pixel has no specific diagnostic information and is thus set to 0 as the background. From the MTI extraction process, the textures in preceding layers have larger importance-values than those in rear layers. This indicates that the textures in preceding layers embody more essential diagnostic information. It can be seen that MTIs can not only describe the edges of ROIs but also can distinguish the significance of these ROIs.

After a MTI is generated, corners are detected on the MTI utilizing the Harris algorithm [11], where the corner response threshold *θ* is discussed in the experiment. Besides storing the coordinate of the corner (*m*, *n*), we also preserve *MTI*(*m*, *n*) as the importance-value of (*m*, *n*).

**Fig. 4:**
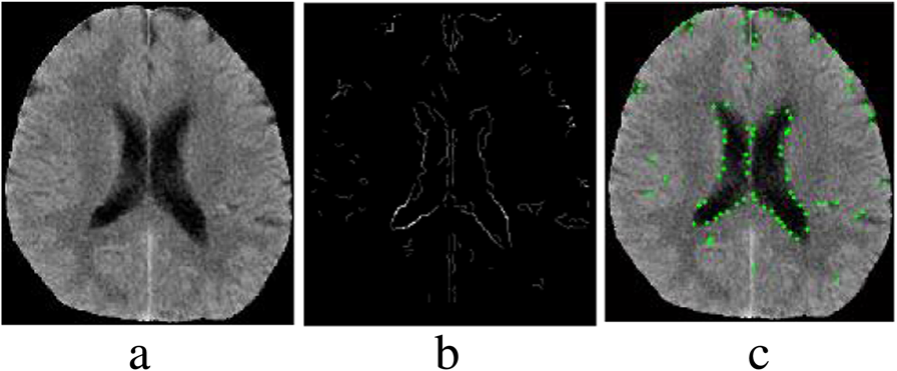
Example of corner detection over a MTI. **a** GI. **b** MTI of the GI. **c** Detected Corners mapped to the GI.

The details of corner detection on a MTI are outlined in Algorithm 1. Line 1-13 describes the idea of MTI extraction, and line 14-21 explains corner detection on the MTI. *F*(*m*, *n*) is the corner response function to determine whether the (*m*, *n*) is a corner or not [11]. Through Algorithm 1, a brain medical image can be represented by a corner sequence *C*. Since the loops in terms of *R* ^*i*^, *m* and *n* are consistent and *s* ^*i*^ can also be solved in consistent steps (*i.e*., less than 64 steps), the time complexity of Algorithm 1 is Ο(1). An example of corner detection on MTIs is demonstrated in Fig. 4.

### Corner matching based on the uncertainty and structure of corners

There exist uncertainty and structure in the pixels of brain medical images [23]. Thus, corners detected from MTIs also remain the uncertainty and structure. In detail, the corners from the same regions among similar brain medical images have similar locations, *i*.*e*., the locations of these corners have certain mobility. Moreover, the mobility of corners is inversely proportional to the importance-values of corners, *i*.*e*., low importance-value indicates high mobility and vice versa. Thus, we define the mobility *Mov*(*m*, *n*) of a corner (*m*, *n*) as below:

3 $$ Mov\left(m,n\right)=\frac{1}{MTI\left(m,n\right)} $$

Based on the mobility of corners, matched corner pairs are generated.

#### **Definition 1**

Given two corner sequences *C* and *C'*, *c*=((*m*, *n*), *MTI*(*m*, *n*))∈*C* and *c*′=((*m*′, *n*′), *MTI'*(*m'*, *n'*))∈*C'*, *c* and *c*′ are defined a matched corner pair [*c*, *c*′] if they satisfy

4 $$ dis\left(c,{c}^{\prime}\right)\le Mov(c)+ Mov\left({c}^{\prime}\right), $$

where *dis*(*c*, *c*′)=||((*m*, *n*), (*m*′, *n*′)||_2_.

Initial matched corner pair sequences are generated based on Definition 1. Nevertheless, the sequences have redundant matched corner pairs. In order to remove the redundancy and meanwhile preserve information to maximum extent, we propose a finial matched corner pair sequence.

#### **Definition 2**

Given an initial matched corner pair sequence *CI*={([*c*_*Ii*_, *c*_*Ij*_′], *dis*(*c*_*Ii*_, *c*_*Ij*_′)) | *c*_*Ii*_∈*C*, *c*_*Ij*_′∈*C*′}, *CM*={([*c*_*Mi*_, *c*_*Mj*_′], *dis*(*c*_*Mi*_, *c*_*Mj*_′)) | *c*_*Mi*_∈*C*, *c*_*Mj*_′∈*C*′} is the corresponding final matched corner pair sequence satisfying: (1) each corner pair [*c*_*Mi*_, *c*_*Mj'*_] is one-to-one; (2) |*CM*| is the maximum; (3) ∑_[*c*__*Mi*_, _*c*__*Mj*_ _′] ∈ *CM*_ *dis*(*c*_*Mi*_, *c*_*Mj*_^′^) is the minimum.
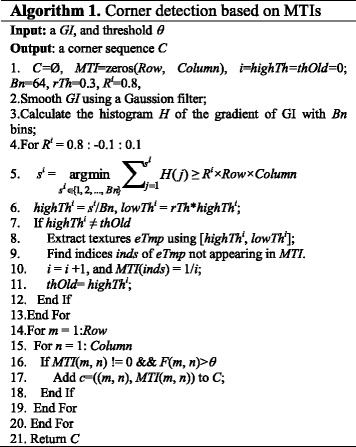
 
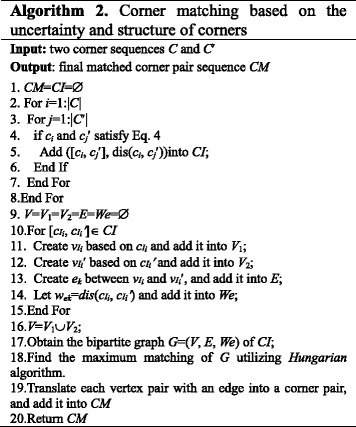


The first case in Definition 2 is to eliminate redundant matched corner pairs. The second and third cases ensure to preserve the longest and most matched corner pairs.

In order to realize *CM*, a biparitite graph *G*=(*V*, *E*, *We*) is generated based on *CI*. The problem of generating the final matched corner pair sequence is transferred to solve the maximum matching of *G*, which has been solved by using the Hungarian method [24, 25].

Algorithm 2 outlines the main ideas of the proposed corner matching method. The vertex number of *G* is (|*C*|+|*C*′|), the maximum edge number of *G* is |*C*|×|*C*′|, and the time complexity of the Hungarian method is Ο(|*C*|×|*C*′|×(|*C*|+|*C*′|)), thus the time complexity of our corner matching is Ο(|*C*|×|*C*′|× (|*C*|+|*C*′|)).

**Fig. 5:**
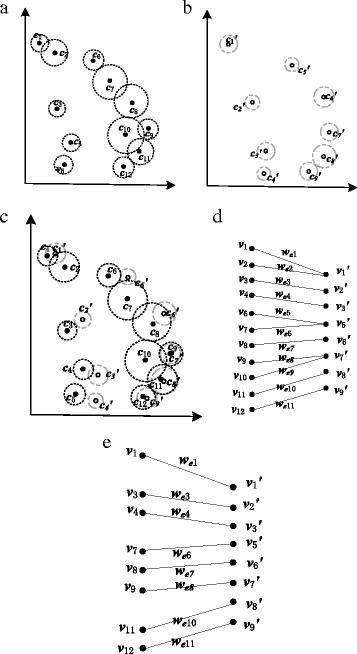
Corner matching example. **a** Corner sequence C **b** Corner sequence C' **c** Overlaying of C and C'. **d** Bipartite graph G based on C and C'. **e** Final matching result of the G

Figure 5 is a corner matching example. Figure 5a and b show two corner sequences C and C′ in a normalized coordinate system, where |C|=12 and |C′|=9 and the dotted circles around these corners reflect their mobility. Figure 5c is the overlapping of C and C′ in the same normalized coordinate system. If the circles around two corners overlap, which means the two corners satisfy Eq. 4, then they are a matched corner pair. Thus, we obtain an initial matched corner sequential CI={([c_1_, c_1_′], dis(c_1_, c_1_′)), ([c_2_, c_1_′], dis(c_2_, c_1_′)), ([c_3_, c_2_′], dis(c_3_, c_2_′)), ([c_4_, c_3_′], dis(c_4_, c_3_′)), ([c_6_, c_5_′], dis(c_6_, c_5_′ )), ([c_7_, c_5_′], dis(c_7_, c_5_′ )), ([c_8_, c_6_′], dis(c_8_, c_6_′ )), ([c_9_, c_7_′], dis(c_9_, c_7_′ )), ([c_10_, c_7_′], dis(c_10_, c_7_′ )), ([c_10_, c_8_′], dis(c_10_, c_8_′)), ([c_11_, c_8_′], dis(c_11_, c_8_′)), ([c_12_, c_9_′], dis(c_12_, c_9_′))}. We can see that CI has redundant matched corner pairs, *i.e*., one-to-many matched corner pairs. For instance, [c_1_, c_1_′] and [c_2_, c_1_′], [c_6_, c_5_′] and [c_7_, c_5_′]. Then a bipartite graph G=(V, E, We) is constructed based on CI as showed in Fig. 5d. In G, V=V_1_∪V_2_, V_1_={v_1_, v_2_, v_3_, v_4_, v_6_, v_7_, v_8_, v_9_, v_10_, v_11_, v_12_}, V_2_={v_1_′, v_2_′, v_3_′, v_5_′, v_6_′, v_7_′, v_8_′, v_9_′}, E={(v_1_, v_1_′), (v_2_, v_1_′), (v_3_, v_2_′), (v_4_, v_3_′), (v_6_, v_5_′), (v_7_, v_5_′), (v_8_, v_6_′), (v_9_, v_7_′), (v_10_, v_7_′), (v_10_, c_8_′), (v_11_, v_8_′), (v_12_, v_9_′)} and We={w_e1_, w_e2_, w_e3_, w_e4_, w_e5_, w_e6_, w_e7_, w_e8_, w_e9_, w_e10_, w_e11_}. Based on the Hungarian method, Fig. 5e shows the maximum matching result of G, *i.e*., the vertex pairs connected by edges. Thus, the final matched corner pair sequence CM = {[c_1_, c_1_′], [c_3_, c_2_′] [c_4_, c_3_′], [c_6_, c_5_′] [c_8_, c_6_′], [c_9_, c_7_′], [c_11_, c_8_′], [c_12_, c_9_′]}.

### Corner-based brain image classification

In two brain medical images, when location of the corners in each matched corner pair is close and the number of unmatched corner pairs is small, the two images tend to be more similar. Based on this, the similarity calculation between brain medical images *I* and *I*′ is proposed based on the *CM*={([*c*_*Mi*_, *c*_*Mj*_′], *dis*(*c*_*Mi*_, *c*_*Mj*_′)) | *c*_*Mi*_∈*C*, *c*_*Mj*_′∈*C*′} of *I* and *I*′, as shown in Eq. 5.

5 $$ SIM\left(I,{I}^{\hbox{'}}\right)=W\left(I,{I}^{\hbox{'}}\right)\times {\sum}_{\left[{c}_{Mi},{c_{Mj}}^{\hbox{'}}\right]\in CM} MD\left({c}_{Mi},{c_{Mj}}^{\hbox{'}}\right) $$

$$ MD\left({c}_{Mi},{c_{Mj}}^{\hbox{'}}\right)=\frac{1}{dis\left({c}_{Mi},{c_{Mj}}^{\hbox{'}}\right)} $$

$$ W\left(I,{I}^{\hbox{'}}\right)=\left\{\begin{array}{c}0,\kern22em \mathrm{if}\kern0.5em \mid C\mid =\mid {C}^{\hbox{'}}\mid \\ {}\begin{array}{l}\frac{1}{\log \left|\left(|C|-| CM|\right)-\Big(|{C}^{\hbox{'}}|-| CM|\Big)\right|}\\ {}=\frac{1}{\log \left|\left(|C|-|{C}^{\hbox{'}}|\right)\right|},\kern13.5em \mathrm{else}\kern1.5em \end{array}\end{array}\right. $$

In Eq. 5, *MD*(*c*_*Mi*_, *c*_*Mj*_′) is the matched degree of [*c*_*Mi*_, *c*_*Mj*_′]. It indicates that the smaller the distance of [*c*_*Mi*_, *c*_*Mj*_′] is, the larger the matched degree of [*c*_*Mi*_, *c*_*Mj*_′] will be. *W*(*I*, *I*′) is a weight, where |*C*| and |*C*′| are the number of corners detected from *I* and *I*′, respectively; |(|*C*|-|*CM*|)-(|*C*′|-|*CM*|)| is the number diversity of the unmatched corners between *I* and *I*′. The value of W(*I*, *I*′) indicates that a small number of the unmatched corners between *I* and *I*′ makes a large effect on the similarity between images.

Based on Eq. 5, *I* can be classified using the KNN model. Specifically, the *K* most similar images of *I* among the training images are selected first. Then *I* is assigned a label whose number among the *K* most-similar images is the largest.

## Results

### Experimental environment

We evaluated the proposed corner-based brain medical image classification method (denoted as the Corner-based method) on the two brain medical image sets: Dct and Dmri. All the experiments were implemented on a computer with an Intel Core (7) processor of 2.60 GHZ and a RAM of 16GB. The development environment includes Eclipse 4.6 and Matlab 2015a.

### Evaluation of the corner-based brain medical image classification

The proposed Corner-based method is evaluated on Dct and Dmri, respectively. First, Dct is randomly divided into two groups of images to generate the training and test image sets. Concretely, 70% of images are separately selected from each of the three types (normal, CI, and CH) of images to form the training set; the remaining 30% of images comprise the test set. In both the training and test sets, the normal type of images are labeled with the “Normal” labels and other two types of images are labeled with the “Abnormal” labels. In this study, the “Normal” category is regarded as positive one and the “Abnormal” category as the negative one. Second, images in the training set are used to construct a classifier. In this process, the 5-folder cross validation approach is employed to measure the performance of the classifier with different *θ* and *K* values. The specific *θ* and *K* with the best experimental performance are chosen to generate the ultimate classifier. Finally, the ultimate classifier is evaluated by predicting the labels of the independent test set. The above steps are repeated on Dmri. Different from Dct, Dmri uses 88% of images as the training set and the others as the test set. The detailed information of the two image sets is summarized in Table 2.

**Table 2:** All brain medical image sets

Image sets	Dct			Dmri	
	Normal	Abnormal		Normal	Abnormal
	normal	CI	CH	normal	AD
training set (training and validation images)	266 (70%)	64 (70%)	20 (30%)	380 (88%)	370 (88%)
test set	114 (30%)	28 (30%)	8 (30%)	50 (12%)	50 (12%)

The performance of the classifiers is evaluated based on four standard indictors: accuracy, precision, recall and F1-score [26]. They are formalized as follows, where TP, FP, TN and FN stand for True Positive, False Positive, True Negative, and False Negative, respectively.

$$ Accuracy=\frac{TP+ TN}{TP+ TN+ FP+ FN} $$

$$ Precision=\frac{TP}{TP+ FP} $$

$$ Recall=\frac{TP}{TP+ FN} $$

$$ F1- score=\frac{2\times precision\times recall}{precision+ recall} $$

**Fig. 6:**
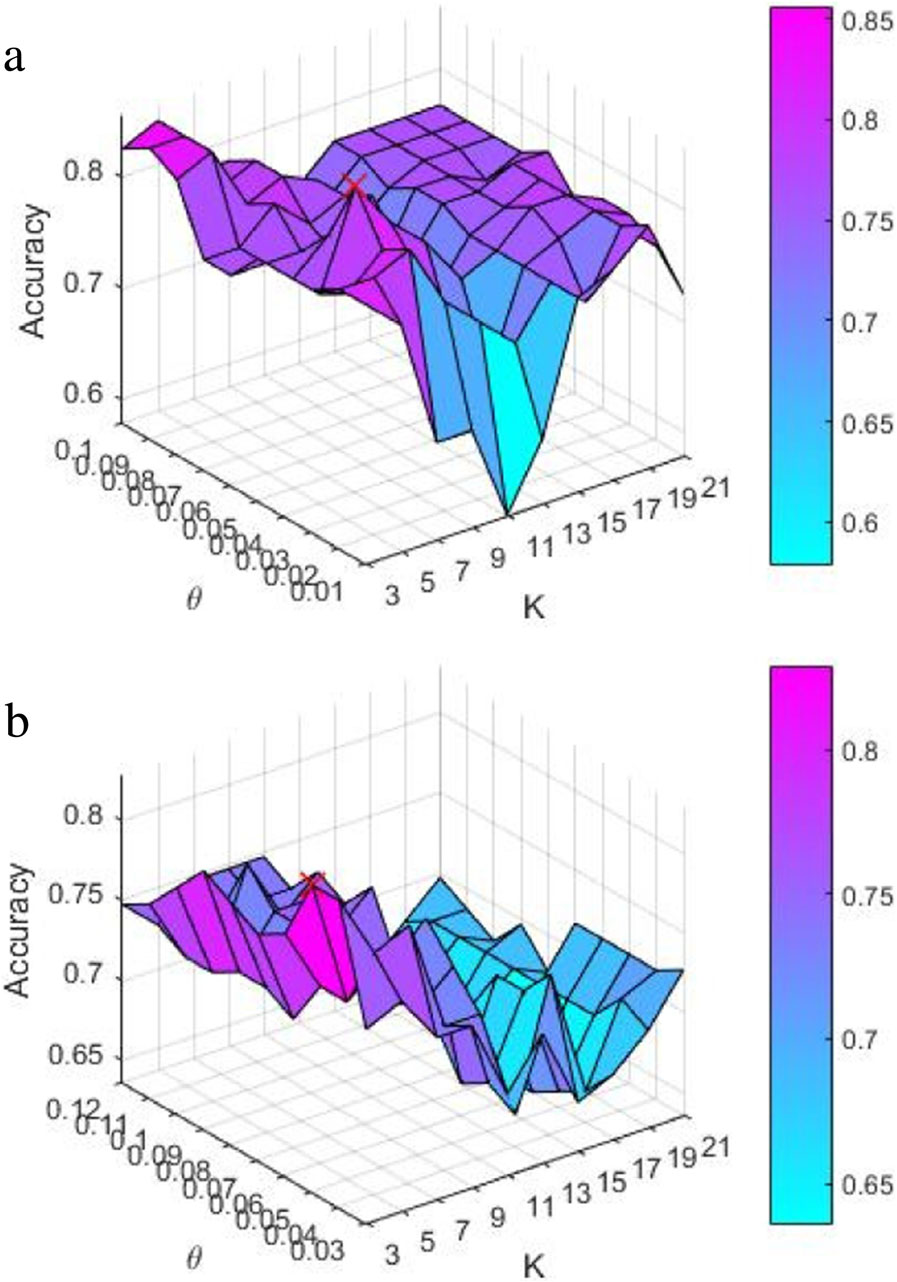
Accuracy of the validation images using the proposed method. *K* is the parameter of the KNN model and *θ* is the corner response threshold for corner detection. **a** Dct. **b** Dmri.

Two parameters, *θ* and *K*, are required to be trained. Specifically, the best *θ* and *K* values are selected based on the accuracy of the validation images to construct classifiers. Figure 6 displays the accuracy of the validation images in Dct and Dmri using the proposed method, respectively. In Dct, *θ* iterates from 0.01 to 0.1 with the growth of 0.01 each time and *K* increases from 3 to 21 with 2 intervals. It can be seen that when *θ*=0.04 and *K*=7, the accuracy reaches the highest point of 0.826 (the red × in the figure). In Dmri, the range of *K* is the same as that in Dct, while the range of *θ* is from 0.03 to 0.12 with the growth of 0.01 each time. It can be seen that *θ* is set to 0.05 and *K* is set to 3 to attain the highest accuracy of 0.828. The two groups of *θ* and *K* are used to construct our two classifiers for Dct and Dmri classification, respectively.

To validate the effectiveness of the proposed method, the method is compared with three baseline brain medical image classification methods: Harris-based method, Texture-based method and Symmetry-based method.

**Fig. 7:**
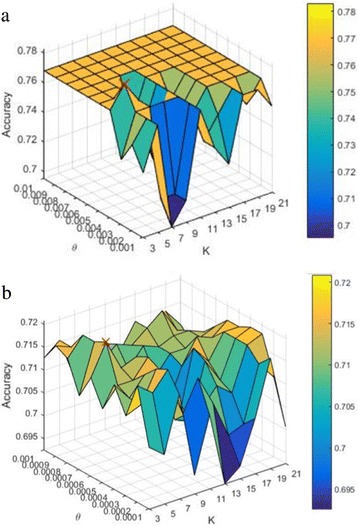
Accuracy of the validation images using the Harris-based method. *K* is the parameter of the KNN model and *θ* is the corner response threshold for corner detection. **a** Dct. **b** Dmri.

The procedure of the Harris-based method is similar to that of the proposed method. It also includes corner detection, corner matching and *θ* and *K* selection for classifiers*.* However, its corners are detected using Harris [11] directly. Since Harris only preserves the coordinates of corners, the importance-values of the detected corners are set to 0 for corner matching. Figure 7 show the accuracy of the validation images in Dct and Dmri using the Harris-based method, respectively. The range of *K* keeps the same. In Dct, *θ* varies from 0.001 to 0.01 with the growth of 0.001 each time. It can be seen that the accuracy reaches the highest point of 0.758 (the red × in the figure) when *θ*=0.04 and *K*=5. In Dmri, *θ* varies from 0.0001 to 0.001 with the growth of 0.001 each time. It can be seen that the highest accuracy is 0.721 when *θ* is set to 0.0006 and *K* is set to 5. The two groups of *θ* and *K* are used to construct two Harris-based classifiers for Dct and Dmri classification, respectively.

The other two comparison methods are based on the methods in [3, 4]. In this study, all the images are 2-D. Since the VBM in [4] is 3-D features, only the texture features are used in the comparison experiment. This method is referred as the Texture-based method. There is no threshold in the Texture-based method according to [4]. In [3], brain medical images are classified into “Normal” or “Abnormal” categories in the first stage by using the symmetry of brain medical images. The method applied in the first stage of [3] is used as another comparison method. It is denoted as the Symmetry-based method. Based on [3], the threshold of the Symmetry-based method is set to 0.88 for brain CT image classification.

We compare the performance of the proposed method with that of the three comparison methods in terms of the test set in Dct. The results are summarized in Table 3. The proposed method is evaluated with *θ*=0.04 and *K*=7 and the Harris-based method is measured with *θ*=0.004 and *K*=5. As can be seen from the table, the proposed method achieves the highest accuracy and F1-score compared with the three comparison methods. This indicates the best performance and good balance of precision and recall of the proposed method. The Harris-based method obtains the best recall, but lowest precision. The Symmetry-based method and the Texture-based method have the leading precision, yet their accuracy, recall and F1-score are all much lower than that of the proposed method.

**Table 3:** Performance of the four comparison methods on Dct

	Proposed method	Harris-based method	Symmetry-based method	Texture-based method
Accuracy	82.6%	75.8%	76.2%	50.0%
Precision	84.4%	76.7%	100%	100%
Recall	94.7%	98.3%	76.2%	50.0%
F1-score	89.3%	86.2%	86.5%	66.7%

The performance of the four comparison methods regarding the test set in Dmri is compared in Table 4. It is noteworthy that *θ*=0.05 and *K*=3 in the proposed method and *θ*=0.0006 and *K*=5 in the Harris-based method. Since the Symmetry-based method was not evaluated on Dmri, we iterated its threshold from 0.01 to 1.0 with the growth of 0.01 at each time. The accuracy reaches the maximum value when the threshold of the Symmetry-based method is between 0.5 and 1.0. It can be seen from the table that the accuracy and F1-score of the proposed method still outweigh that of the three comparison methods. In addition, the recall of the proposed method is also the highest. Though the precision of the Symmetry-based method is larger than that of the proposed method, the other measurements of the Symmetry-based method fall behind that of the proposed method greatly. The results also indicate that the proposed method can achieve the best comprehensive performance on Dmri.

**Table 4:** Performance of the four comparison methods on Dmri

	Proposed method	Harris-based method	Symmetry-based method	Texture-based method
Accuracy	78.2%	70.9%	65.1%	50.0%
Precision	92.2%	91.8%	95.3%	0%
Recall	72.3%	65.0%	49.4%	NaN
F1-score	81.0%	76.1%	65.1%	NaN

Table 5 displays the runtime of the four comparison methods when they perform on the test sets of Dct and Dmri. The number in the front of / denotes the entire runtime of testing Dct or Dmri and the rear number denotes the average runtime of testing Dct or Dmri. It can be seen that in both Dct and Dmri, the trends in runtime among the four methods are the same. The proposed method is less efficient than the Symmetry-based method yet more efficient than both the Harris-based method and the Texture-based method. Though the efficiency of the proposed method is not as good as that of the Symmetry-based method, its average runtime for classifying each brain CT and MRI image is 1.73s and 0.61s, respectively. Thus, the proposed method is still acceptable.

**Table 5:** Runtime(s) of the four comparison methods on Dct and Dmri

	Proposed method	Harris-based method	Symmetry-based method	Texture-based method
Dct	260/1.73	387/2.58	26/0.17	3104/20.69
Dmri	61/0.61	214/2.14	2/0.02	1590/15.90

## Discussion

In this study, we develop a robust corner-based brain medical image classifier. First, we propose a corner detection method combing diagnostic information from neurologists. This method consists of MTI extraction and corners detection on MTIs. Second, we present the definition of matched corner pairs and put forward a bipartite model to generate the final matched corner pair sequence. Finally, we propose a similarity calculation method based on the matched and unmatched corner pairs for brain medical image diagnosis. We demonstrate that the proposed corner-based classifier can efficiently achieve the highest accuracy and F1-score for brain medical image classification and is robust to the intensity ranges of brain medical images caused by different brain imaging modalities.

Based on the priori diagnostic information that different regions in brain medical images have diverse diagnostic information, we extract MTIs. Specifically, we utilize multiple groups of thresholds to separately extract the multilayers of textures from different regions and assign these textures different significance to form MTIs. Then, corners are detected on MTIs and are assigned specific importance-values based on the corresponding significance in MTIs. Thus, these detected corners can not only describe the morphology of brain medical images but also demonstrate their specific significance. Moreover, since brain medical images have the uncertainty and structure, corners also inherit these characteristics. The uncertainty makes corners have mobility, yet the structure limits the movable ranges of corners. Specifically, the movable ranges of significant corners are relative small yet the movable ranges of insignificant corners are comparative large. Based on the movable ranges and importance-values of corners, we define a matched corner pair to produce an initial matched corner pair sequence. We further propose a bipartite graph to reduce the redundancy of the initial matched corner pair sequence and generate the final matched corner pair sequence for classification. The proposed method takes advantage of the priori diagnostic information and the characteristics of brain medical images. This makes it achieve great performance and robust to different intensity ranges of brain medical images.

Different from the Texture-based method [4] and Symmetry-based method [3], both the proposed method and the Harris-based method utilizes corners as features to classify brain medical images. Moreover, compared with the Harris-based method, the proposed method assigns corners importance-values by employing the priori diagnostic information. In Dct, the proposed method outperforms the three comparison methods in terms of accuracy and F1-score. The Harris-based method achieves better accuracy and F1-score than the Texture-based method and attains competitive accuracy and F1-score compared with the Symmetry-based method. In Dmri, both the proposed method and the Harris-based method gain higher accuracy and F1-score compared with the Texture-based method and the Symmetry-based method. The proposed method still outweighs the Harris-based method. These results indicate that (1) corners are discriminative features for both brain CT and brain MRI image classification; (2) employing the diagnostic information and the characteristics of brain medical images enhances the performance of the proposed method. These results are because corners are the key points of the morphology of images and the morphology of organs or lesions in brain medical images are the main evidence of doctors’ diagnosis. Though textures can reflect the morphology of images, the Texture-based method regards the textures of different regions equally while the proposed method distinguish the significance of the textures of different regions. Moreover, in terms of the proposed method, its accuracy and F1-score on Dmri decrease 4.4% and 8.3% compared with that on Dct. In terms of the Harris-based method, its accuracy and F1-score on Dmri decrease 4.9% and 10.1% compared with that on Dct. In terms of the Symmetry-based method, its accuracy and F1-score on Dmri decrease 11.1% and 21.4% compared with that on Dct. In terms of the Texture-based method, its F1-score on Dmri decreases 66.7%; though its accuracy keep the same, the accuracy values on both Dct and Dmri are 50%, which is a random value in binary classification. These results indicate that (1) the corner-based methods (*i.e*., the proposed method and the Harris-based method) are more robust to different intensity ranges of brain medical images caused by the different imaging modalities in this study (the maximum intensity value of Dct is 256 and the Dmri’s is 65536); (2) employing the diagnostic information in the proposed method enhances the robustness compared with the Harris-based method. These are because of the following reasons: during the process of MTI extraction based on the diagnostic information, different layers of textures are assigned different importance-values to reflect the diagnostic information. These importance-values are calculated based on the histogram of an image’ gradient values not an image’s intensity values. Since corners are detected on MTIs, this alleviates the dependence of the proposed corner detection method on the intensity values of images. Thus, the proposed method can endure the variability of the intensity ranges caused by different brain imaging modalities in this study. Furthermore, the proposed method is more efficient than the Harris-based method and Texture-based method yet less efficient than the Symmetry-based method. However, the proposed method does not use any optimization, which will be handled in our future work.

## Conclusion

In this study, we first propose a corner detection method combining the diagnostic information of brain medical images. It consists of two steps: MTI extraction and corner detection based on MTIs. These detected corners are assigned different importance-values to distinguish their significance. Second, we put forward a corner matching method. It produces an initial matched corner pair sequence based on the uncertainty and structure of brain medical images and further generates a final matched corner pair sequence via a bipartite graph model. Finally, a similarity function is proposed based on the final matched corner pair sequence and is used to make diagnoses for brain medical images. Experimental results on brain CT and MRI medical image sets show that the proposed corner-based brain medical image diagnosis method outperforms the three comparison methods, and is more robust to the intensity ranges of brain medical images caused by different brain imaging modalities.

Recent studies have shown multimodal medical images can provide complementary information to enhance diagnostic accuracy. In the future, we will focus on corner detection and matching based on the multimodal brain medical images. Moreover, the proposed method does not take parallel computing into account, we will boost the efficiency of the proposed method in a parallel way.
